# Why QRS Duration Should Be Replaced by Better Measures of Electrical Activation to Improve Patient Selection for Cardiac Resynchronization Therapy

**DOI:** 10.1007/s12265-016-9693-1

**Published:** 2016-05-26

**Authors:** Elien B. Engels, Masih Mafi-Rad, Antonius M. W. van Stipdonk, Kevin Vernooy, Frits W. Prinzen

**Affiliations:** 1 Department of Physiology, Cardiovascular Research Institute Maastricht, Maastricht University, Maastricht, The Netherlands; 2 Department of Cardiology, Maastricht University Medical Center, Maastricht, The Netherlands

**Keywords:** Electrocardiography, Vectorcardiography, Cardiac mapping, Cardiac resynchronization therapy, Left bundle-branch block

## Abstract

Cardiac resynchronization therapy (CRT) is a well-known treatment modality for patients with a reduced left ventricular ejection fraction accompanied by a ventricular conduction delay. However, a large proportion of patients does not benefit from this therapy. Better patient selection may importantly reduce the number of non-responders. Here, we review the strengths and weaknesses of the electrocardiogram (ECG) markers currently being used in guidelines for patient selection, e.g., QRS duration and morphology. We shed light on the current knowledge on the underlying electrical substrate and the mechanism of action of CRT. Finally, we discuss potentially better ECG-based biomarkers for CRT candidate selection, of which the vectorcardiogram may have high potential.

## Introduction

Cardiac resynchronization therapy (CRT) is an effective therapy for patients with a decreased left ventricular ejection fraction (LVEF) in combination with a ventricular conduction delay, especially due to left bundle-branch block (LBBB). CRT creates a more coordinated and efficient contraction of the heart, improves LV systolic function and quality of life, and reduces heart failure (HF) symptoms, hospitalizations, and mortality [1, 2].

Nevertheless, there is still an incomplete understanding of the mechanism of the therapy and unsatisfying selection of patients. On the one hand, a significant portion (30–50 %) of patients that are implanted according to current guidelines [3, 4] benefit little from this therapy whereas ∼20 % of patients show complete normalization of LVEF [5]. Possible explanations for this huge range of benefit are variation in substrate that is amenable to resynchronization, inadequate device settings, suboptimal medical treatment, arrhythmias, and variable lead position [6].

The most important selection criteria in current CRT implantation guidelines are derived from the electrocardiogram (ECG): QRS duration and morphology [3, 7]. Here, we review the strengths and weaknesses of these ECG markers in the light of the current knowledge on the underlying electrical substrate and the mechanism of action of CRT and discuss potentially better ECG-based biomarkers for selection of CRT candidates.

## The Role of the 12-Lead ECG in the Selection of CRT Candidates

The clinical application of CRT began in 1994 when the first cases of atrio-biventricular pacemaker implantations in patients with severe congestive HF were described [8, 9]. The surface ECG of these patients often showed a prolonged PR interval and a widened QRS complex due to ventricular conduction disturbances.

The first randomized crossover trial investigating the clinical efficacy of CRT was the Multisite Stimulation in Cardiomyopathy (MUSTIC) study [10]. This trial in patients with chronic severe HF (New York Heart Association (NYHA) III), reduced LVEF (<35 %) and a broad QRS complex (>150 ms), showed that biventricular (BiV) pacing improved the 6-min walking distance, peak oxygen uptake, quality of life score, and NYHA class. The multi-center insync randomized clinical evaluation (MIRACLE) study confirmed these results in patients with a QRS duration ≥130 ms [2, 11]. This study also showed a clear reduction in LV volumes, reduced HF hospitalization, and better survival. Similar results were shown by the COMPANION [12] and the cardiac resynchronization (CARE)-HF [1] trials, which included patients with QRS duration ≥120 ms and NYHA class III–IV.

These favorable and consistent results led to the recommendation of CRT in patients in NYHA class III–IV despite optimal medical treatment, with a reduced LVEF (<35 %), in sinus rhythm, and a wide QRS complex (≥120 ms) [13].

**Fig. 1:**
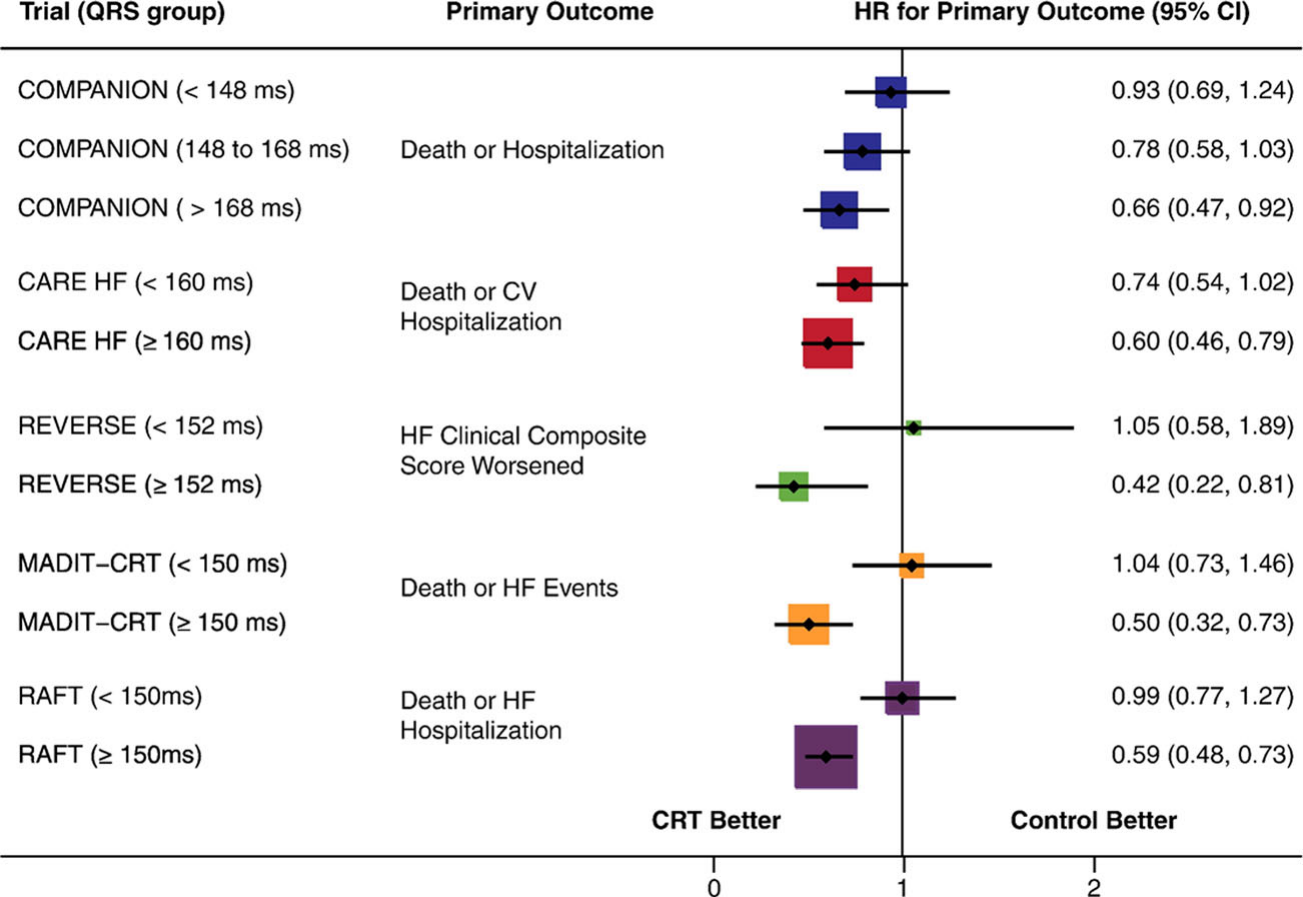
Effect of CRT on composite clinical events in patients with moderately prolonged (QRS duration of 120–150 ms) and severely prolonged QRS duration (>150 ms) (reprinted from [17])

Subsequent trials investigated the effect of CRT in less symptomatic patients (the resynchronization reverses remodeling in systolic left ventricular dysfunction (REVERSE) [14], multicenter automatic defibrillator implantation trial (MADIT)-CRT [15], and resynchronization/defibrillation for ambulatory heart failure trial (RAFT) trials [16]). Again, LV function improved, and both all-cause mortality and non-fatal HF events improved. However, subgroup analyses of these three trials demonstrated that these effects were predominantly confined to patients with a QRS duration ≥150 ms (Fig. 1) [17]. This evidence resulted in the addition of a class I indication to CRT for patients presenting with NYHA class II, a reduced LVEF, and a QRS duration >150 ms, in the 2010 guidelines [18].

Even though most studies show an increased response rate after CRT in patients with a severely prolonged QRS duration, these studies used the fairly crude division of the cohorts in patients with a QRS duration < and >150 ms. However, the best cutoff value for QRS duration is unclear.

**Fig. 2:**
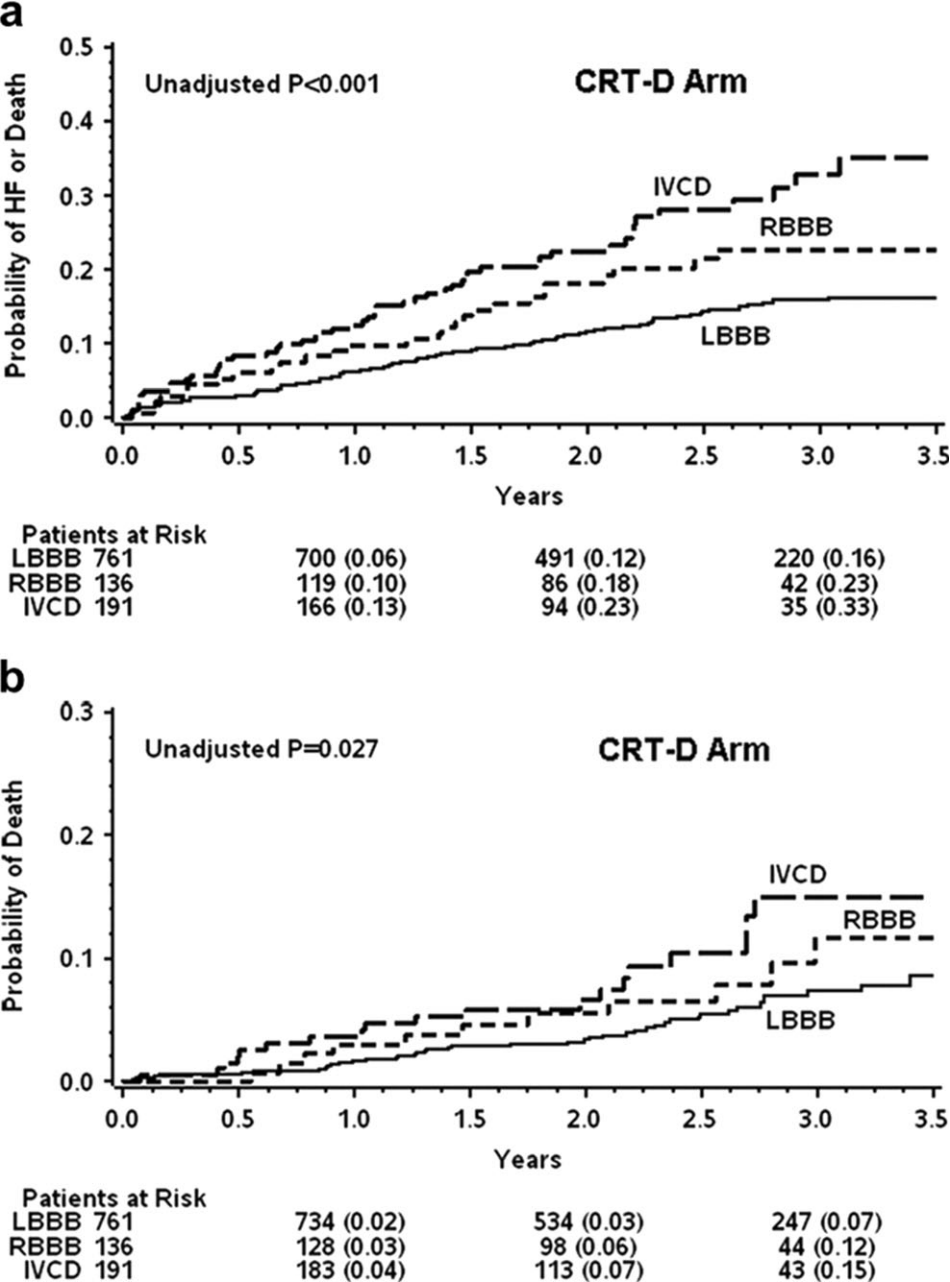
Cumulative probability of HF event or death **a** and of death alone **b** according to QRS morphology in the CRT with defibrillator (CRT-D) arm of the MADIT-CRT (adjusted from [21])

More recently, attention has shifted from QRS duration to QRS morphology. Small single-center studies [19, 20] and sub-analyses of the MADIT-CRT [21], REVERSE [22], and RAFT [16] study showed that patients with a LBBB morphology benefit most from CRT. In contrast, patients with right bundle-branch block (RBBB) or intra-ventricular conduction delays (IVCD) had no benefit or even a worse outcome from CRT (Fig. 2). These observations led to the adaptation of the guidelines in 2012/2013, including LBBB as the primary ECG criterion and QRS duration >150 ms only if a non-LBBB morphology is present [3, 4].

Interesting and important, however, is that the definition of complete LBBB from the 12-lead ECG varies between European and American guidelines and between large clinical trials [21, 22] or studies [23] that investigated LBBB as a predictor of CRT effectiveness. The refinement of LBBB morphology with the presence of notching or slurring appears to significantly improve the prediction of CRT response and clinical outcome, at least in small single-center studies [20, 24].

While QRS morphology is now one of the primary indicators for CRT, a recent meta-analysis, combining data from CARE-HF, MIRACLE, MIRACLE ICD, REVERSE, and RAFT showed that QRS duration is a more powerful predictor of CRT outcomes (mortality and morbidity) than QRS morphology [25]. This conclusion is in contrast to several reports derived from some of the individual trials and to a meta-analysis of the MADIT-CRT, RAFT, and REVERSE study (Fig. 1) [26]. One possible explanation for this discrepancy is the use of “liberal” LBBB criteria. In that case, it is likely that QRS duration provides additional information. Indeed, when using “liberal” LBBB criteria the non-LBBB patients tended to have a lower QRS duration than the LBBB patients [21], but this difference could not be observed when stricter LBBB criteria were used [20]. Furthermore, in the studies where strict LBBB criteria as defined by Strauss et al. [23] were used, QRS duration was not a predictor of response while LBBB was [20, 27].

In conclusion, currently it is not clear whether QRS duration or morphology should be preferred as primary marker for selection of CRT patients. QRS duration may not be specific, but LBBB criteria may be too complex and/or dependent. In order to come to a possible solution, it may be worthwhile to go back to the basic physiology of dyssynchronous HF and the mechanisms of CRT.

## Electrophysiological Evaluation of the Electrical Substrate for CRT

Delayed electrical activation of the LV is considered the underlying substrate of LV dysfunction in patients with systolic dysfunction and a conduction delay, mainly due to LBBB [28]. CRT aims to correct the underlying electrical substrate by paced pre-excitation of late depolarized and contracting LV regions, thereby restoring synchronous ventricular electrical activation and contraction [28]. Experimental studies have confirmed that in hearts with delayed LV activation due to LBBB, LV-only or BiV pacing creates a more synchronous contraction pattern, which is accompanied by marked hemodynamic improvement [28, 29]. The clinical importance of LV activation delay has become evident in studies showing that a greater delay in time from onset of the QRS complex to the local intrinsic activation at the LV stimulation site (Q-LV) is associated with a greater likelihood of benefit from CRT. Singh et al. measured Q-LV intra-procedurally as a percentage of the baseline QRS interval in 71 patients undergoing CRT device implantation [30]. A longer Q-LV was related to superior acute LV hemodynamic improvement, whereas a reduced Q-LV (<50 % of QRS duration) was related to a worse clinical outcome [30]. A secondary analysis of the prospective multi-center SMART-AV trial showed that patients with a Q-LV > 95 ms show significantly improved odds of reverse remodelling and quality of life response [31]. Conversely, experimental studies and computer simulations have shown that pacing induced pre-excitation in a heart without a significant electrical delay (narrow QRS complex) widens the QRS complex and consequently worsens LV pump function [32–34]. The clinical significance of these findings has become evident in the results of the recent EchoCRT trial [35]. This was a randomized trial that evaluated the effect of CRT in patients with a narrow QRS complex (<130 ms) and evidence of mechanical dyssynchrony. The trial was prematurely stopped because the CRT group did not derive any detectable clinical benefit and even showed a significant increase in mortality compared to the control group [35].

All the aforementioned data support the notion that an electrical substrate, consisting of a sufficient amount of LV activation delay, needs to be present for CRT to be efficient. LBBB is considered the hallmark conduction disturbance that is associated with delayed LV activation. In canine hearts where proximal ablation of the left bundle-branch was performed, electrical mapping showed that earliest electrical activation occurs inside the right ventricle and that the electrical wave front then slowly propagates through the interventricular septum towards the lateral wall of the LV [36]. Induction of LBBB in healthy canine hearts leads to electrical and mechanical dyssynchrony that in turn causes loss of LV pump function and ventricular remodelling [37]. In these hearts, CRT largely reverses functional and structural abnormalities [28]. The key clinical investigation to detect and evaluate the extent of LV activation delay remains the surface ECG. However, identifying true LBBB on the ECG is not as straightforward as one might presume. As discussed earlier, numerous dissimilarities in ECG criteria for the diagnosis of LBBB between different definitions complicate a uniform diagnosis.

The most accurate way to evaluate the cardiac electrical activation sequence in patients is by invasive mapping using conventional point-by-point technique or three-dimensional electro-anatomical reconstruction contact (CARTO, NOGA) or non-contact (EnSite) mapping. Studies that performed endocardial mapping in patients with HF and LBBB according to conventional ECG criteria have shown that the sequence of LV endocardial activation in these patients is heterogeneous [38–41]. The activation wave front originating from the right ventricle was shown to cause LV endocardial breakthrough in different septal regions [39, 40]. In some patients, breakthrough occurred in the vicinity of the conduction system in the mid-septal region, which suggests activation by slow conduction through the left bundle-branch, in others, LV endocardial activation occurred as a result of right-to-left transseptal spread of activation [40]. A characteristic finding in true LBBB patients also seems to be a long (>40 ms) transseptal conduction time [42].

Endocardial non-contact mapping has also identified two different patterns of electrical wave front propagation in the LV of these patients. The first entity, observed in approximately two thirds of patients, is characterized by a U-shaped pattern of activation that turns around the LV apex and inferior wall in order to activate the lateral wall [39, 41, 43], which is similar to the activation pattern that has been observed during endocardial non-contact mapping in canine hearts where proximal ablation of the left bundle-branch has been performed [44]. The second entity is characterized by homogeneous propagation of electrical activation throughout the left ventricle [41, 43]. The varying conduction patterns observed in these mapping studies could be explained by variations in left bundle-branch anatomy [45] and the location of the block, but also by the fact that cellular uncoupling as a consequence of LV hypertrophy or fibrosis can give rise to a wide QRS complex with morphological features that meet conventional ECG criteria for LBBB [46, 47].

In contrast to LBBB, RBBB is typically associated with delayed RV activation, but not delayed LV activation. However, in some RBBB patients, the QRS morphology differs significantly from the characteristic RBBB pattern. These patients show a specific electrocardiographic pattern previously defined as RBBB masking LBBB [48, 49], which is characterized by precordial lead findings consistent with RBBB and limb lead findings consistent with LBBB. Extensive measurements of both RV and LV endocardial electrical activation in heart failure patients with RBBB using CARTO 3D contact mapping showed that patients with RBBB masking LBBB have LV activation delay similar to that found in LBBB [50].

**Fig. 3:**
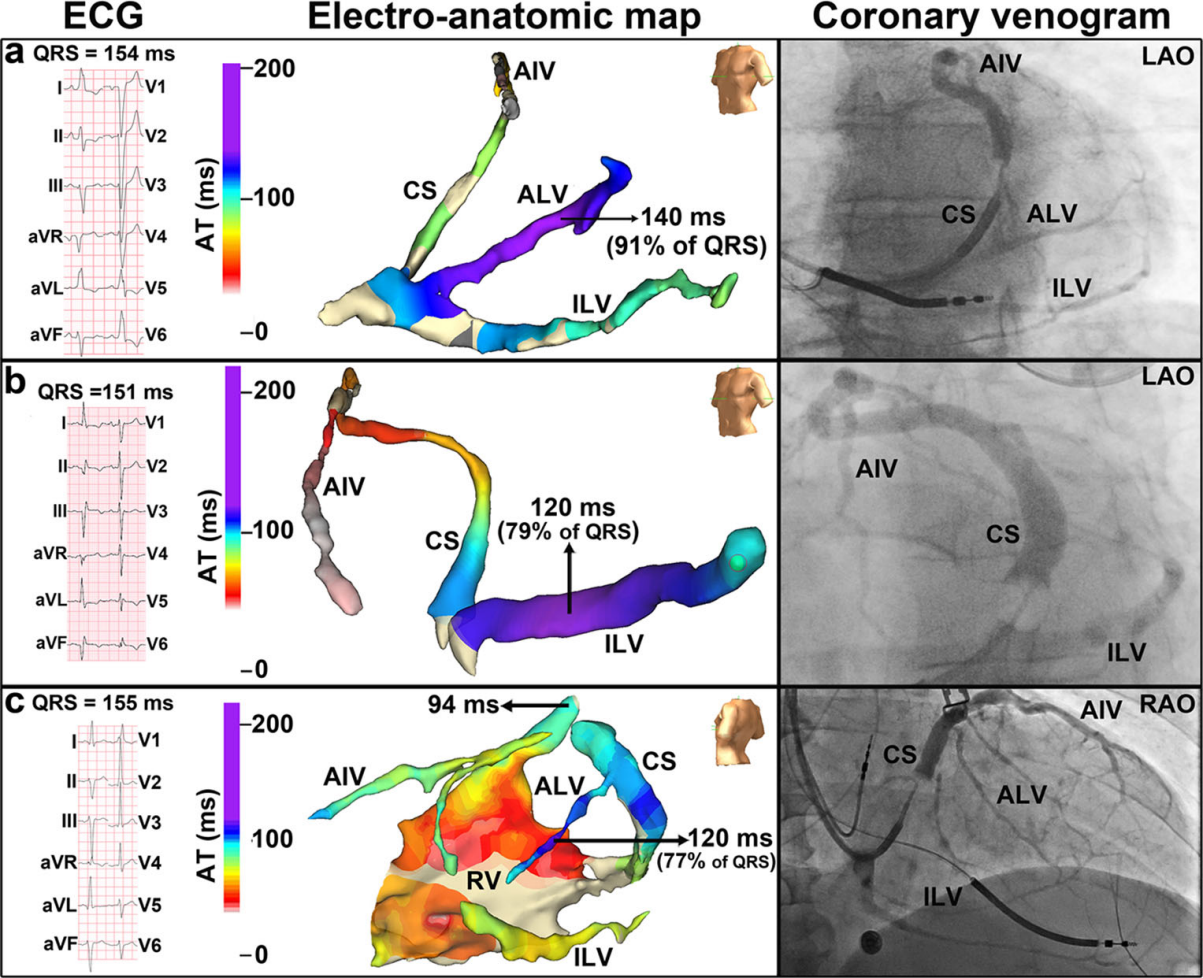
Coronary venous electro-anatomic map of a LBBB patient demonstrating delayed activation of the LV anterolateral wall **a**, an IVCD patient demonstrating delayed activation of the LV inferolateral wall **b**, and a RBBB patient with a potential left anterior hemiblock demonstrating delayed activation of the LV anterolateral wall **c**. *AIV* anterior inter-ventricular vein, *ALV* anterolateral vein, *ILV* inferolateral vein, *CS* coronary sinus, *AT* electrical activation time, *AP* antero-posterior, *L/RAO* left/right anterior oblique, *RV* right ventricle (adapted from [51])

Although the aforementioned mapping techniques provide accurate characterization of cardiac electrical activation, the application of these techniques in clinical practice is time-consuming, cumbersome, and not without risk. Measuring the Q-LV as described above provides a relatively simple manner of assessing the extent of LV activation delay. However, this technique provides limited information on LV electrical activation because usually measurements are only performed at the anatomically targeted region. A technique that provides a middle ground between complete mapping and single Q-LV measurement is intra-procedural coronary venous electro-anatomic mapping. In a recent study, we assessed the LV electrical activation in a cohort of 51 CRT candidates using this technique [51]. A guidewire that allows for unipolar sensing and pacing was inserted into the coronary sinus and connected to an EnSite NavX system. The wire was then manipulated to various coronary sinus branches creating an anatomic map along with determining the electrical activation time associated with each anatomic region. Significant LV activation delay (>75 % of QRS duration) was found in 38 of 51 patients. QRS duration was shown to perform poorly in identifying delayed LV activation (area under the curve = 0.49). Twenty-nine of the 51 patients had LBBB according to specific ECG criteria which included broad, notched, or slurred R waves in leads I, aVL, V5, and V6, an occasional RS pattern in leads V5 and V6 attributed to displaced transition of the QRS complex, and absent q waves in lead I, V5, and V6 (in the absence of a large anterior-apical infarction). As described earlier, this refined LBBB definition, which includes the presence of QRS notching and slurring, has previously been shown to significantly improve the predictive value of LBBB QRS morphology for CRT response [52]. Of the remaining 22 patients, 7 met ECG criteria for RBBB and 15 met neither criteria for LBBB nor RBBB and were classified as IVCD. QRS duration did not differ between different QRS morphologies. However, LV activation time was significantly larger in LBBB patients as compared to RBBB and IVCD patients. Significant LV activation delay was found in all patients diagnosed with LBBB according to specific ECG criteria, but also in 8 of 15 patients with IVCD and even in 1 of 7 patients with RBBB (examples shown in Fig. 3). The findings of this mapping study indicate that (1) a prolonged QRS duration by itself is not a reliable marker of delayed LV activation. Thus, patient selection based on QRS duration alone will most likely include a substantial number of patients without the appropriate electrical substrate to benefit from CRT, and (2) the refined LBBB definition, which includes QRS notching and slurring, is highly specific for delayed LV activation, but lacks sufficient sensitivity. As a consequence, a substantial number of patients that have delayed LV activation are not identified as such, and in these patients, CRT may be withheld erroneously.

Instead, the above described technique of coronary venous electro-anatomic mapping can be used at the time of CRT implantation for a more precise characterization of the electrical substrate at only minor prolongation of procedure time (∼20 min) [51, 53]. However, ideally the decision whether or not to implant a CRT device is made in advance. In this respect, electrocardiographic imaging (ECGi) provides an entirely non-invasive alternative [54]. ECGi provides high-resolution non-invasive electrical mapping of the epicardial electrical activation. The technique acquires electrical data from more than 200 body surface electrodes using a multi-electrode vest. Epicardial anatomy and body-surface electrode positions are registered simultaneously by a thoracic computed tomography scan. The body-surface electrical data and the anatomical data are then processed with algorithms to construct epicardial depolarization and repolarization patterns, using a single heartbeat [54]. In this way, detailed information on LV electrical activation can be readily obtained prior to CRT implantation, which may be used to guide the decision on whether or not to implant a CRT device. However, the requirement for a multi-electrode vest in combination with a computed tomography scan may preclude widespread application of this technique in clinical practice.

## Better Electrocardiographic Identification of the Electrical Substrate: New ECG Parameters

**Fig. 4:**
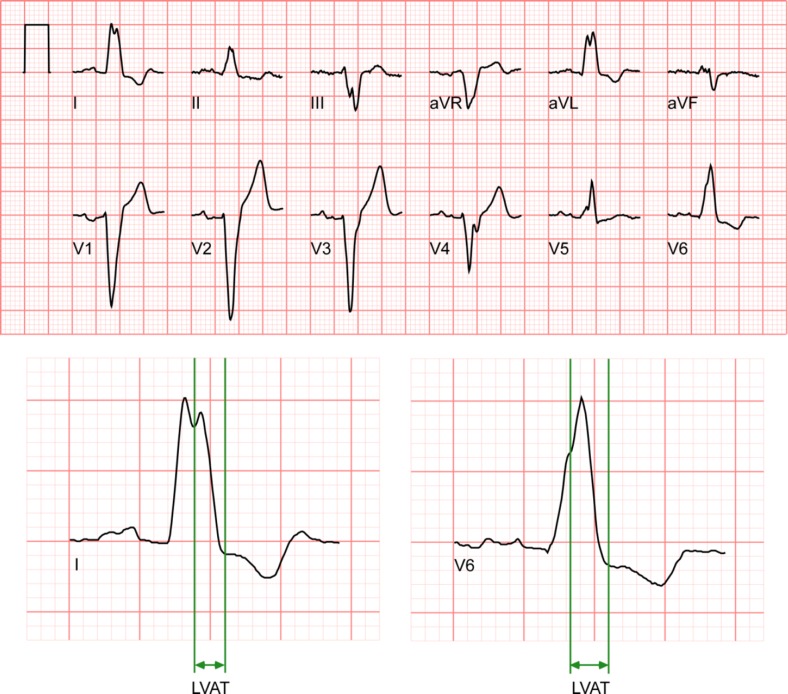
Example of a left ventricular activation time (LVAT) measurement. LVAT_max_ is measured as the time difference between the first notch after 40 ms of QRS onset and the end of the QRS

The demand for easy and widely applicable non-invasive techniques that can be used to accurately characterize the electrical substrate in CRT candidates has renewed the interest in finding additional/alternative electrocardiographic markers of dyssynchrony. Sweeney et al. carefully analyzed standard 12-lead ECGs of 202 CRT candidates with LBBB according to specific ECG criteria that included QRS notching/slurring and identified new measurements that predict volumetric CRT response [19]. The time difference between the first notch after 40 ms of QRS onset and the end of the QRS on the baseline ECG was indicated as the LV activation time (LVAT_max_, Fig. 4). A longer LVAT_max_ was shown to be predictive of CRT response (OR[CI] = 1.30[1.11–1.52] for each 10 ms increase up to 125 ms). In addition, the Selvester QRS score for LBBB was used to quantify LV scar extent. A higher Selvester score was negatively associated with reverse remodelling (OR[CI] = 0.49[0.27–0.88] for each 1-point increase from 0 to 4; 0.92[0.83–1.01] for each 1-point increase >4) [19].

Recently, the value of the vectorcardiogram (VCG) for characterizing the electrical substrate and predicting CRT response has been explored. VCG is a technique that records the magnitude and direction of the electrical forces that are generated by the heart over time, resulting in a resultant electrical force depicted by a vector for each time point. Connecting the arrow heads of all vectors, a vector loop is constructed. The VCG thus contains 3D information of the electrical forces within the heart, which might provide more valuable information than the 1D-ECG. It was hypothesized that large electrical dyssynchrony, amenable to CRT, would lead to large unopposed electrical forces during ventricular depolarization and that the size of these forces may be well represented by the QRS_AREA_, the area of the QRS complex in the three principle directions. Van Deursen et al. assessed the area of the QRS complex (QRS_AREA_) on the VCG in 81 consecutive CRT candidates and showed that a large QRS_AREA_ was associated with high odds of long-term volumetric CRT response. Moreover, QRS_AREA_ predicted CRT response better than QRS duration and then conventionally defined LBBB and as least as good as the most refined LBBB definition [24].

**Fig. 5:**
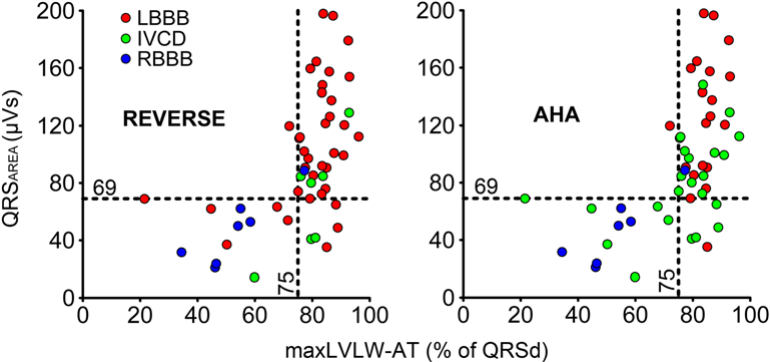
QRS_AREA_ plotted as a function of maximal LV lateral wall activation time (maxLVLW-AT) expressed as percent of QRS duration (QRSd) for all patients (each *dot* represents a patient, *n* = 51) with LBBB diagnosed according to the definition provided by the REVERSE trial (*left*) and the American Heart Association (AHA) definition (*right*). This figure demonstrates the excellent diagnostic performance of QRS_AREA_ >69 μVs for delayed LV lateral wall activation (defined as a maxLVLW-AT exceeding 75 % of QRS duration), independent of the QRS morphology on the surface ECG, and illustrates the difference in QRS morphology classification caused by disparity in LBBB definitions (adapted from [51])

The notion that QRS_AREA_ represents the extent of unopposed electrical forces is supported by the observation that QRS_AREA_ is larger in patients with LBBB as compared to patients with IVCD and that QRS_AREA_ is lower in ischemic than in non-ischemic patients [24]. Further support comes from observations in the abovementioned study on coronary venous mapping. In this study, VCGs were constructed from pre-procedural standard 12-lead ECGs for all patients using the Kors algorithm. A large QRS_AREA_ (>69 μVs) on the VCG was shown to be highly predictive of delayed LV lateral wall activation as determined by coronary venous mapping (Fig. 5) [51]. On the other hand, QRS_AREA_ has been shown to be smaller in patients with heart failure of ischemic etiology, which may be explained by the presence of non-conductive fibrotic tissue [24]. Taken together, these observations suggest that QRS_AREA_ is not only useful to determine the extent of electrical dyssynchrony, but that it may also reflect the presence of determinants known to reduce the chance of CRT benefit, such as an ischemic etiology of heart failure. However, more research is required to better understand all determinants of QRS_AREA_.

**Fig. 6:**
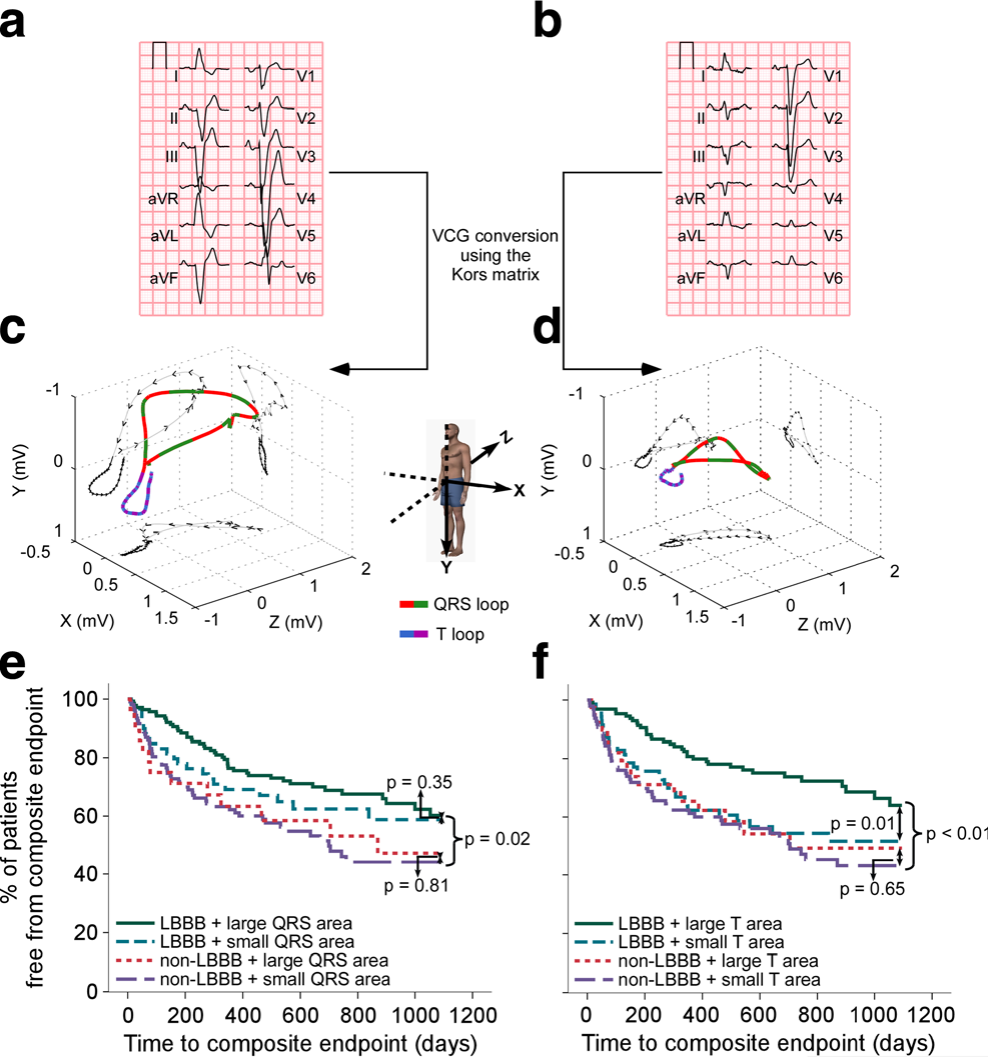
Typical example of VCGs constructed from standard 12-lead ECGs for a patient with a large **a**, **c** and a patient with a small **b**, **d** T-wave area, despite being both classified as having LBBB. Panels **e** and **f** show Kaplan-Meier estimates of the probability free from the composite endpoint HTLD (HF hospitalization, heart transplantation, LVAD implantation, death) after 3 years of CRT. Large QRS or T area are values ≥median value and small QRS or T area are values <median value (adapted from [55, 56])

Interestingly, two studies showed that VCG-derived measures of repolarization predict CRT response even better than QRS_AREA_. Engels et al. assessed the T-wave area from VCGs of 244 CRT recipients (VCG examples shown in Fig. 6c, d). The VCG-derived T-wave area was shown to predict echocardiographic CRT response better than QRS_AREA_ [55]. In a larger cohort consisting of 335 CRT recipients in which the primary endpoint was the composite of heart failure hospitalization, heart transplantation, left ventricular assist device implantation, or death during a 3-year follow-up period, the predictive power of T-wave area for CRT response was found to be primarily evident in the group of patients with LBBB (Fig. 6e, f) [56]. A large T-wave area in LBBB patients was associated with less HF hospitalizations and a higher chance of survival [56]. The size of the T-wave area is a reflection of the extent of unopposed electrical forces during the repolarization phase. The T-wave area is partially determined by the size of the QRS_AREA_ [55], but other factors such as changes in K^+^ and Ca^2+^ ion channel expression might also play a role. In this study, a larger T-wave area was primarily caused by a larger amplitude and not so much by a longer JT-interval. Further research is needed to investigate which other factors are exactly reflected in the T-wave area.

A limitation of all these studies regarding the QRS_AREA_ is that relatively small sample sizes were used. Furthermore, the studies related to the prediction of CRT response using the QRS_AREA_ were all retrospective. Therefore, these results need to be validated in a larger prospective study.

The great practical benefit of QRS_AREA_ and T-wave area is that these parameters are measured in an objective manner and quantified as continuous variables, as opposed to LBBB which is a dichotomous measurement that is subject to the use of different definitions and subjective interpretations of QRS notching/slurring. Another practical feature of QRS_AREA_ and T-wave area is that they can easily be derived from the standard 12-lead ECG. Most commercially available ECG machines have algorithms to construct VCGs from standard 12-lead ECGs using the inverse Dower or Kors’ regression transformation [57, 58]. These VCGs provide a good resemblance of the gold standard Frank VCG and have recently also been validated for use in patients with dyssynchronous heart failure [59]. The non-invasive and simple nature of VCG analysis combined with the excellent predictive power of QRS_AREA_ and T-wave area for CRT response indicates that these parameters can be easily applied in clinical practice to identify appropriate candidates for CRT, thereby potentially improving response to this therapy.

## Conclusion

Based on the evidence obtained from electro-anatomic mapping that QRS_AREA_ reflects LV activation delay, the primary electrical substrate for CRT, and on the better prediction of CRT response by QRS_AREA_ as compared to QRS duration, we propose to include QRS_AREA_ in the guidelines as a selection criterion for CRT implantation. The possibly even better prediction of CRT response by using the T-wave rather than the QRS complex requires further investigation.

## Abbreviations

LV: Left ventricle
EF: Ejection fraction
LBBB: Left bundle-branch block
CRT: Cardiac resynchronization therapy
HF: Heart failure
ECG: Electrocardiogram
ECGi: Electrocardiographic imaging
BiV: Biventricular
